# Management of Tuberculosis of the Hip With Antitubercular Therapy and Single-Stage Total Hip Replacement: A Case Report

**DOI:** 10.7759/cureus.65110

**Published:** 2024-07-22

**Authors:** Rahul Singh, Suhas Landge, Saksham Goyal, Sachin Goel, Ankit R Upadhyay

**Affiliations:** 1 Department of Orthopaedics, Jawaharlal Nehru Medical College, Datta Meghe Institute of Medical Sciences, Wardha, IND; 2 Department of Orthopaedics, Jawaharlal Nehru Medical College, Datta Meghe Institute of Higher Education and Research, Wardha, IND

**Keywords:** infectious disease, tubercular arthritis, total hip replacement (thr), antitubercular therapy (att), hip tuberculosis

## Abstract

Hip tuberculosis (TB) is not a common disease, and this devastating illness requires complete treatment. This case study describes the treatment of a 25-year-old female who suffered from hip TB. She came with right hip discomfort, limping, and a restricted range of movement. The clinical examination showed a fixed flexion deformity, adduction, internal rotation, and leg shortening. Radiographic imaging showed arthritis with hip joint space narrowing, bone erosion, and bone disintegration. Laboratory testing revealed increased inflammatory markers, and a synovial fluid investigation showed tuberculous arthritis. The initial treatment consisted of a regular four-drug antitubercular therapy (ATT) regime for six months and then an additional four months of isoniazid and rifampicin. This therapy resulted in improved clinical symptoms and decreased inflammatory markers. However, the level of joint degeneration required surgical intervention. Due to substantial joint damage, the patient received a hybrid total hip replacement (THR) after completing ATT, confirming that the infection had been cured. Intraoperative observations included synovial enlargement, bone erosions, and significant cartilage damage. The patient underwent a rehabilitation program following surgery to regain mobility and hip joint range of motion. The patient reported substantial pain relief and functional improvement at the one-year follow-up with no signs of implant loosening or infection recurrence.

## Introduction

Tuberculosis (TB) affecting the hip joint, although less prevalent compared to spinal TB, remains a significant source of morbidity, particularly in regions with higher TB burdens, which are predominantly found in developing countries [1,2]. Management of this condition necessitates a comprehensive approach involving medical and surgical interventions adapted to the stage of disease progression and the degree of joint involvement [3,4].

Osteoarticular TB is typically caused by the hematogenous spread of *Mycobacterium tuberculosis* bacilli originating from a primary focus, such as the lungs or lymph nodes. These bacteria can then settle in the synovium or bone, forming granulomas, caseation necrosis, and eventual destruction of the joint [5,6]. Timely diagnosis and initiating antitubercular therapy (ATT) are critical for preserving joint function and preventing further deterioration [7,8]. In advanced stages, surgical interventions like joint debridement or arthroplasty may become necessary to alleviate pain and enhance mobility [9,10].

Total hip arthroplasty (THA) is a viable treatment choice in cases where TB of the hip has been adequately treated and the infection has cleared. As illustrated by a growing number of global reports, the placement of metal implants in tuberculous infections has proven safe and excellent. THA is a consideration when a patient experiences debilitating end-stage arthritis/ankylosis (joint stiffness) of a hip joint related to a prior tuberculous infection. In cases where the hip joint has been significantly impaired, resulting in considerable discomfort and disability, THA offers a potential alternative to reduce symptoms and regain functional mobility [11,12].

## Case presentation

Clinical presentation

**Figure 1:**
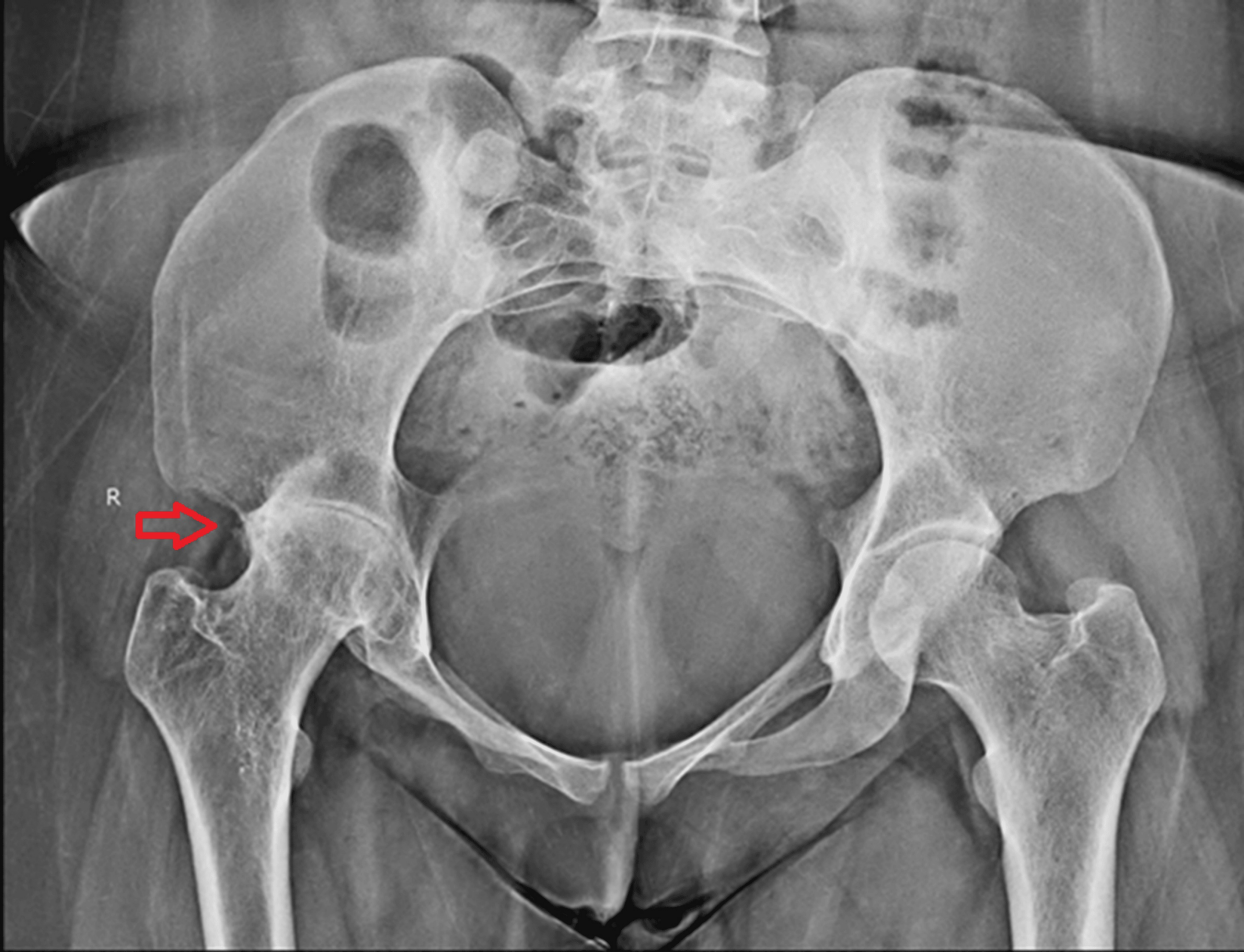
The arrow shows the right hip with advanced arthritis and joint space narrowing, erosions, and bone destruction, consistent with the advanced stage of tuberculous arthritis.

A 25-year-old female patient presented with a one-year history of progressive right hip pain, limping, and restricted movements. The patient is from India, a region where tuberculosis is endemic. Her past medical history includes pulmonary tuberculosis, but there is no history of previous exposure to tuberculosis or trauma. Clinical examination revealed a flexion deformity, adduction, internal rotation of the right hip, and apparent limb shortening. The patient reported significant discomfort with weight-bearing and experienced difficulty performing daily activities. Laboratory investigations revealed elevated erythrocyte sedimentation rate (ESR) of 65 and C-reactive protein (CRP) levels of 120, indicating an active inflammatory process. Synovial fluid aspiration was sent for AFB and were consistent with tubercular finding, further supporting the clinical suspicion. Radiographic findings demonstrated advanced arthritis with joint space narrowing, erosions, and bone destruction, consistent with the advanced stage of tuberculous arthritis, as shown in Figure 1.

**Figure 2:**
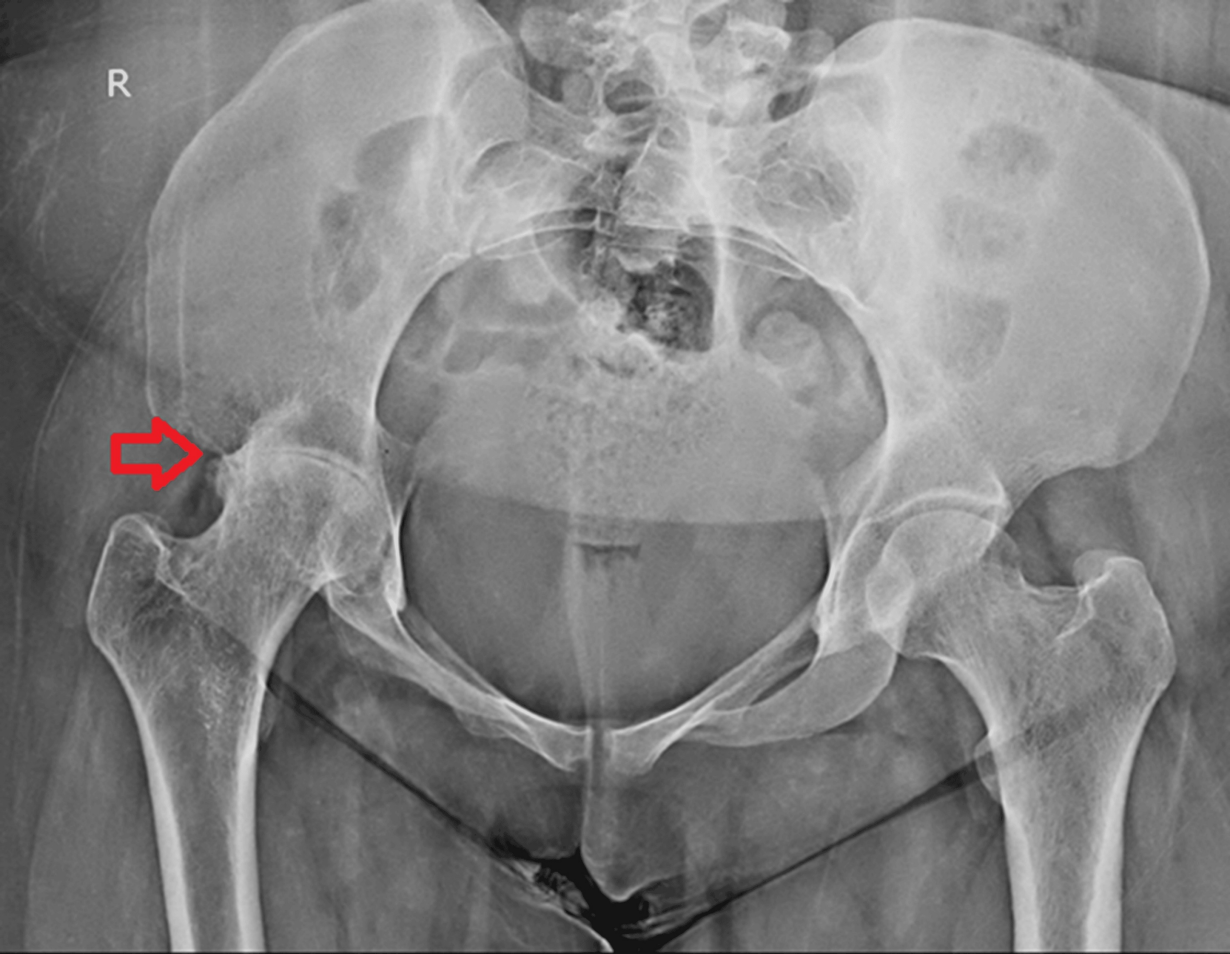
Follow-up radiograph after 12 months of ATT shows no further advancement of arthritic changes in the right hip joint. ATT: anti-tubercular therapy

She was started on anti-tubercular therapy and was advised to follow up regularly, and a repeat X-ray was done on follow-up, as shown in Figure 2.

MRI findings

**Figure 3:**
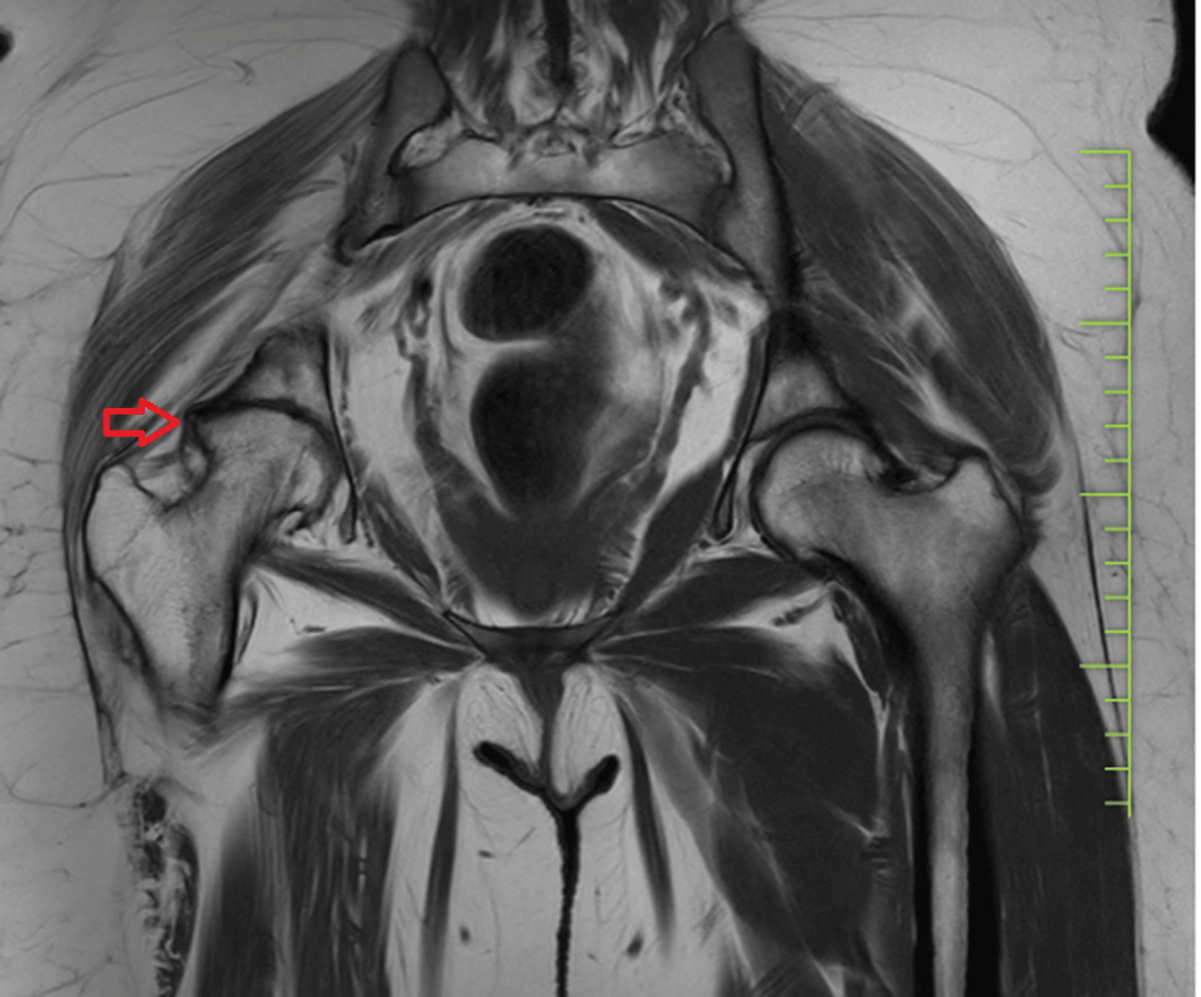
MRI of the pelvis with both hip coronal sections showing osteoarthritis of the right hip with reduced joint space and erosion of acetabular and femoral head surfaces. MRI: magnetic resonance imaging

**Figure 4:**
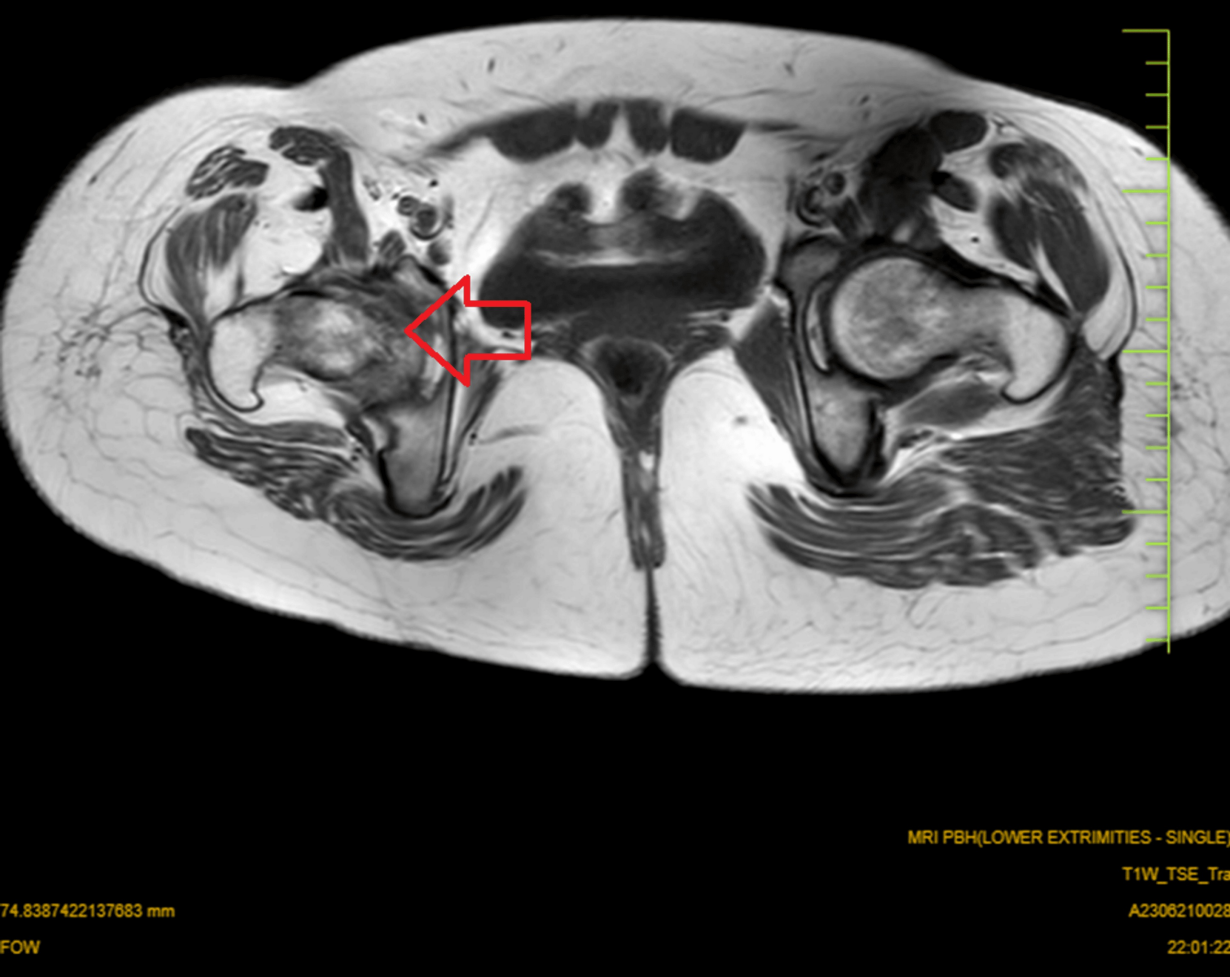
MRI of the pelvis with both hip axial sections shows reduced joint space on the right side as compared to the left side. MRI: magnetic resonance imaging

Magnetic resonance imaging (MRI) of the affected hip joint revealed extensive synovial hypertrophy, bone marrow edema, and areas of bone erosion and destruction, further confirming the advanced stages of the disease, as shown in Figures 3-4.

Based on the clinical presentation, laboratory findings, radiographic evidence, and MRI findings, a diagnosis of advanced tuberculous arthritis of the right hip, was established, and the patient was started on an anti-tubercular regimen for 12 months.

**Figure 5:**
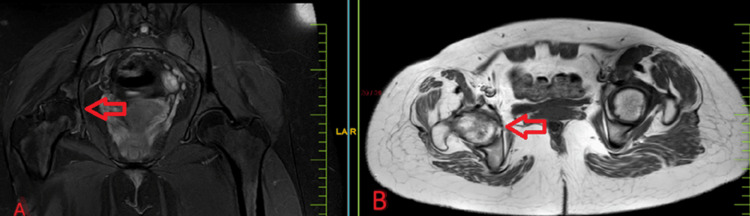
MRI pelvis with the hip A) coronal section and B) axial section showing that the right femoral head is seen slightly impacted in the left acetabulum causing surface irregularity of both the femoral head and acetabular cavity with loss of sphericity. Reactive edema is also seen in the acetabulum, and a crescent sign is noted in the right femoral head. These features suggest avascular necrosis of the right femoral head and Ficat and Arlet stage III classification. MRI: magnetic resonance imaging

A repeat MRI was done after a year, and no signs of infection were seen. The MRI showed avascular necrosis of the right femoral head, grade III Ficat-Arlet grading, as shown in Figure 5A-B.

Management

After completing ATT laboratory investigations, they revealed a standard limit of ESR and CRP levels, indicating no active inflammatory process. Hence, the patient was planned for a single-stage hybrid (cemented cup and uncemented stem) THA.

The surgical procedure posterior approach was taken, and following complete curettage and resection of the infected tissue, various samples were obtained and sent for microbiological and histological examinations. The microbiological examinations included acid-fast bacilli (AFB) staining, which is a crucial step in detecting the presence of mycobacterial organisms. With no signs of active infection, a decision was made to continue with THA, and hence, a cemented cup and uncemented stem were done in this case.

Postoperative Care and Management

Vigilant surveillance is essential in instances of THA for TB of the hip to promptly identify any possible recurrence or reactivation of the infection maintaining a high level of clinical suspicion while assessing the potential presence of pyogenic infections or a reoccurrence of TB is essential. Regular evaluation is needed, encompassing the observation of clinical symptoms and inflammatory markers, and radiological imaging examinations should be done, which will assist in the efficient monitoring and treatment of this patient.

Postoperative Rehabilitation

Postoperative rehabilitation and mobilization procedure for a THA performed for tubercular involvement of the hip joint is not materially different from the protocol used for THAs for other reasons. Hence, she was started with early mobilization and walking as soon as she was comfortable after the surgery. To achieve the best functional results, she was advised to follow a supervised physiotherapy program that includes static exercises and aided mobility with suitable walking aids. A thorough strategy for postoperative and mobilization is crucial in facilitating healing and regaining mobility.

**Figure 6:**
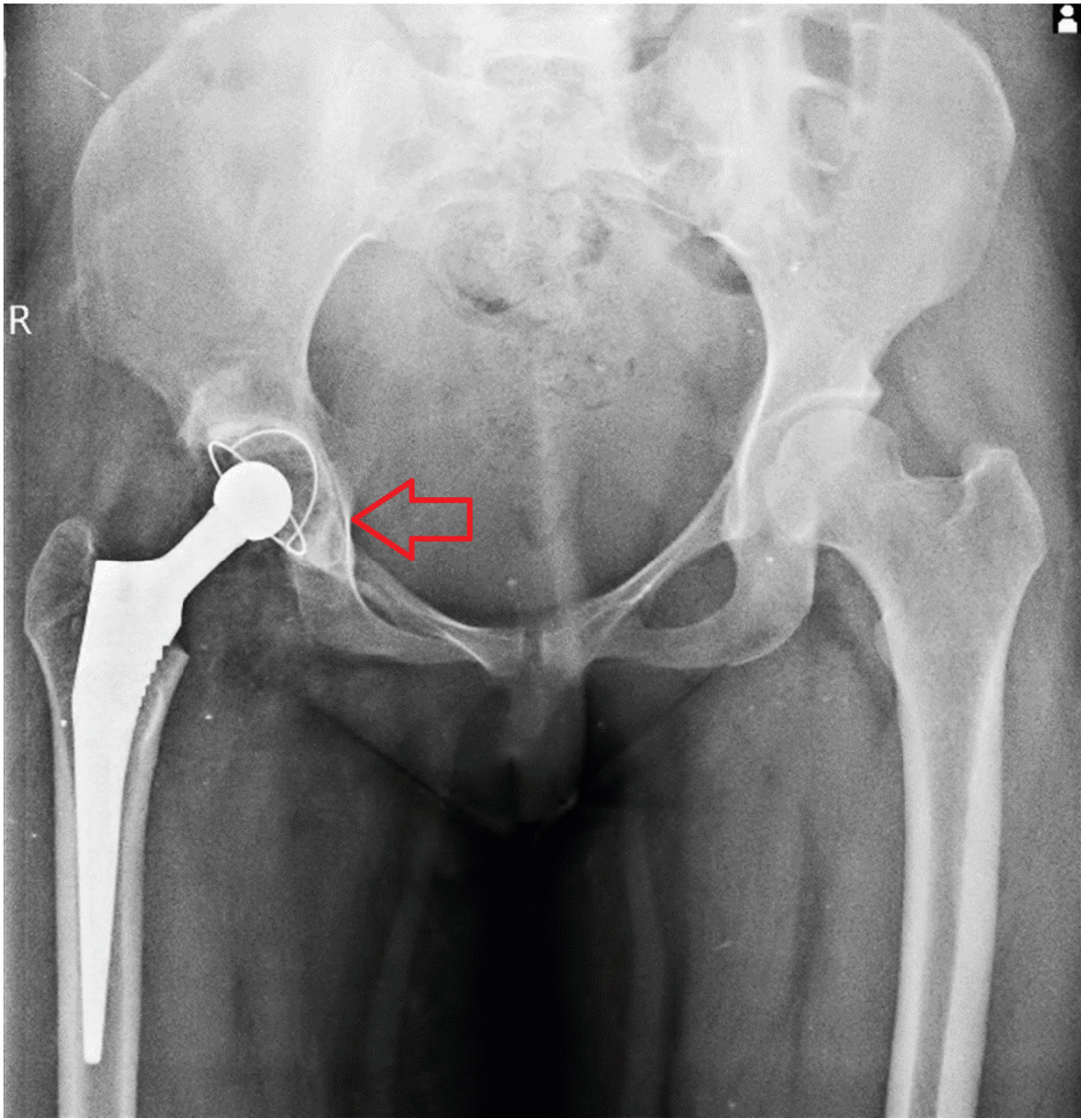
Postoperative follow-up radiograph of the pelvis with both hips' anteroposterior view showing stable total hip arthroplasty prosthesis in situ.

A postoperative radiograph with both hips showed stable THA prosthesis in situ, as shown in Figure 6.

## Discussion

This case report underscores the successful management of advanced tuberculous arthritis of the right hip through a judicious combination of ATT and THR surgery. Several key factors facilitated the favorable outcome. Detecting tuberculous arthritis at an early stage is crucial. It allows for the prompt initiation of ATT, which is essential in controlling the infection and preventing extensive joint damage. Early intervention helps preserve joint function and improves the overall prognosis of the condition [1,5]. ATT is the cornerstone of managing tuberculous arthritis. Prompt initiation of ATT can significantly reduce the bacterial load, control the infection, and prevent the spread of TB to other joints and organs. This step is critical in managing the disease and ensuring a successful outcome [3,10]. In cases of advanced tuberculous arthritis with significant joint destruction, surgical intervention in total hip replacement becomes necessary. The timing of this surgery is crucial. Performing the surgery too early may not allow sufficient time for ATT to control the infection while delaying surgery can lead to further joint damage and complicate the procedure. Therefore, a well-timed surgical intervention after adequately controlling infection with ATT is imperative [13]. During THR surgery, meticulous debridement of the infected tissue is necessary to reduce the bacterial load and create a clean environment for the implant. This step helps minimize the risk of postoperative infections and improves the longevity and functionality of the joint replacement [1,5].

Postoperative rehabilitation is a critical component of recovery. Adherence to a structured rehabilitation program helps restore joint function, improve mobility, and ensure the long-term success of the joint replacement. Rehabilitation protocols should be tailored to the patient's needs and involve a multidisciplinary team of physiotherapists and rehabilitation specialists [13]. The management of tuberculous arthritis of the hip necessitates a multidisciplinary approach involving the expertise of orthopedic surgeons, infectious disease specialists, and rehabilitation professionals. This collaboration ensures comprehensive care, from diagnosis and medical management to surgical intervention and postoperative rehabilitation [3]. However, long-term follow-up studies are warranted to establish the safety and durability of joint replacements in the setting of active or healed tuberculous arthritis.

This case report contributes to the growing body of evidence supporting the role of THR in managing advanced tuberculous arthritis of the hip when performed in conjunction with appropriate medical treatment and surgical debridement. The successful outcome in this case highlights the importance of a multidisciplinary approach, early diagnosis, prompt initiation of ATT, and judicious timing of surgical intervention in managing tuberculous arthritis of the hip. It underscores the need for continued research and long-term followup studies to understand further and optimise the treatment protocols for this challenging condition [13].

## Conclusions

Managing tuberculous arthritis of the hip necessitates a multidisciplinary approach that integrates ATT, surgical debridement, and, in advanced cases, joint replacement surgery. Early diagnosis, coupled with the prompt initiation of ATT, emerges as paramount to preserving joint function and staving off further destruction.

For cases in advanced stages characterized by significant joint damage, THR stands as a viable option. When complemented by sufficient ATT and surgical debridement, THR offers relief from pain and facilitates functional improvement and the restoration of mobility.

By combining these interventions, clinicians can effectively address both the infectious and structural aspects of tuberculous arthritis of the hip, thereby enhancing patient outcomes and quality of life. THA should be performed on patients with good immunity under pre- and postoperative antitubercular drugs.
